# The association between Long-COVID symptomology, perceived symptom burden and mental health in COVID-19 patients in Shijiazhuang, China: a population-based health survey

**DOI:** 10.3389/fpsyt.2024.1332066

**Published:** 2024-01-26

**Authors:** Yufei Li, Lawrence T. Lam, Ying Xiao, Zhengqi Qiu, Yanming Zhang

**Affiliations:** ^1^Faculty of Medicine, Macau University of Science and Technology, Macao, Macao SAR, China; ^2^Guangdong-Hong Kong-Macau Joint Laboratory for Contaminants Exposure and Health, Guangzhou, Guangdong, China; ^3^Hospital-Acquired Infection Control Department, Sanya Central Hospital, Sanya, Hainan, China

**Keywords:** Long-COVID, depression, anxiety, stress, mental health, post-COVID conditions

## Abstract

**Background:**

Long-COVID (LC) refers to post-acute COVID-19 symptoms that can last for months or longer after the initial infection, affecting the physical health of infected patients. This study aims to investigate the association between the symptomology of LC and the mental health of patients in China. It also aims to examine the relationship between the perceived symptom burden and mental health of these patients.

**Methods:**

A population-based stratified cluster sample was recruited, using a standard sampling procedure, from a prefecture-level city in Northern China. Participants included patients who had tested positive for COVID-19 after December 2022. LC symptomology was assessed using a LC symptoms checklist where the perceived symptom burden was measured by the included 5-point Likert scales. Mental health of patients was measured using the Depression, Anxiety, and Stress Scale (DASS), the original Connor-Davidson Resilience Scale (CD-RISC), and the Duke-UNC Functional Social Support Questionnaire (DUFSS). Data were analysed using multiple linear regression models.

**Results:**

About 25% of respondents, experienced COVID symptoms lasting longer than two months that could only be explained by the infection. Post-exertional malaise (22.2%) and fatigue (21.2%) were the most common symptoms. After controlling for potential confounding variables, LC symptomology was significantly and positively associated with depression (t=2.09, p=0.037) and anxiety (t=4.51, p<0.001), but not stress. Perceived symptoms burden was also positively and significantly related to depression (β=0.35, p<0.001), anxiety (β=0.54, p<0.001), and stress (β=0.35, p<0.001), suggesting a dose-response relationship between perceived symptom burden and mental ill health.

**Conclusion:**

This study highlights the importance of recognising the risk of LC, patients’ perception of the symptom burden and its potential impact on mental health. Healthcare professionals should be aware of the complexity of psychological comorbidities among infected patients reporting prolonged symptoms, and be able to give advice regarding long-term management of the symptoms.

## Introduction

The COVID-19 pandemic has not only caused almost 800 million cases and 7 million deaths globally, but also the long-lasting and profound changes at the personal level, such as social interactions, lifestyles, and mental-wellbeing. It also affected the population at the social level including the global economy, healthcare system, and social inequalities (1, 2). In October 2021, the World Health Organization (WHO) defined Long-COVID (LC) as the condition typically occurs in individuals with a history of probable or confirmed SARS-CoV-2 infection, usually 3 months after onset, lasting for at least 2 months and cannot be explained by any alternative diagnosis (3). With global numbers of infections of COVID-19 exceeding 500 million, a conservative estimate of people being currently affected by LC worldwide could be more than 100 million (4). Typical acute COVID-19 cases are characterised by respiratory symptoms, fever, and neurological symptoms (5). The duration and severity of acute COVID-19 cases vary, with some patients being asymptomatic, while others require hospitalisation and mechanical ventilation. The average duration of a COVID-19 infection is typically shorter than 4 weeks (6). The new-onset conditions including cardiovascular, thrombotic/cerebrovascular disease, type 2 diabetes, myalgic encephalomyelitis/chronic fatigue syndrome (ME/CFS) and dysautonomia, especially postural orthostatic tachycardia syndrome (POTS), could last for a long time even years. These symptoms could be commonly called LC. It also have different labels, such as post-acute sequelae of COVID-19, post-acute COVID-19, or post-COVID-19 condition (7).

It has been widely reported that the risk of psychiatric symptoms was associated with COVID-19 infection. A recent meta-analysis revealed that up to one in four patients experienced neuropsychiatric symptoms spanning sleep disorders, fatigue, anxiety, and post-traumatic stress (PTSD) after the onset of COVID-19 with an approximate follow-up duration of 77 days (8). Studies have further explored potential correlations between the risk of LC and mental health problems. Specifically, LC was found to be associated with increased display of depression, anxiety, PTSD, and weakened life satisfaction (9), which might be partially attributed to the persistent physical symptoms of LC (10). In China, few studies have focused on evaluating the impact of LC on COVID survivors. Zhao et al.’s study (11) found that approximately 9.6% of hospitalised patients suffering from moderate impairment reported mainly mental health and cognitive symptoms 20 months after recovery. Several factors were reported to be predictive of long-term physical and cognitive symptoms, which included age, hospital stay, sex, and comorbidities. However, the sample was recruited in 2021 and was not based on a randomised sampling method. Another study conducted in China has found that having at least one LC symptom increased the risk of depression or anxiety by 3.44-fold (12). However, the study sample was limited to hospitalised patients recruited in 2020. Given the limitations of these studies, there is a need to further study the mental health effect of LC, particularly in patients who were infected in the later period of the pandemic using a random community-based sample.

The extent of suffering from chronic illness is not just determined by the severity of the illness itself, but rather moderated by external and subjective factors (13). As The Burden of Treatment Theory posits, management of chronic conditions involves routine work for patients to control their illness. As treatment burdens accumulate, some patients become overwhelmed leading to poorer outcomes, stress on caregivers, and increased healthcare costs (14). In addition, personality factors also play an important role when patients cope with the illness. According to Leventhal’s self-regulatory model, patients’ “illness representations” (beliefs about cause, timeline, consequences, etc.), reflecting their perceived burden, affect their self-management behaviors, psychological adjustment, and mental health (15). Therefore, the perceived burden of illness of patients suffering from Long-COVID, like any other chronic conditions, may also have a direct impact on their mental health.

In terms of the relationship between the perceived severity of LC symptoms and the mental health of patients, few studies have been conducted. In the study by Sivan and colleagues, it was found a positive correlation between the perceived severity of 12 types of LC symptoms and cognitive symptoms, PTSD, depression, and anxiety (16). However, in another study of patients with LC symptoms, the perceived severity of autonomic dysfunction and anxiety, as well as emotional well-being were not positively correlated (17).

Therefore, this study aims to describe the common symptoms related to LC and to quantify the prevalence of these symptoms among community-dwelling COVID patients. It also aims to examine the relationship between the LC symptomology and the mental health status of these patients, as well as the relationship between the perceived burden of the LC symptoms and the level of mental health problems. It is hypothesised that LC symptomatology is associated with the mental health of patients and that there is a positive dose-response relationship between the perceived burden of LC symptomatology and the severity of mental health problems.

## Materials and methods

### Settings and participants

This was a population-based cross-sectional health survey using a stratified cluster random sample recruited from Shijiazhuang, a prefecture-level city in Northern China. Since residents of the city were housed in compounds in China, two compounds from the list of compounds within each jurisdiction in the city were randomly selected in accordance with the population size of jurisdictions. In total, eight compounds were selected as the sample frame. Two buildings were then randomly selected from each compound for the recruitment of the sample. Additional buildings will be selected if the sample size does not reach the expected level. The researchers joined the WeChat group of the selected buildings to distribute the online questionnaire with the permission of the compound’s management. The online survey was designed using the Tencent platform which could be easily distributed through social media platforms. For participants who were not familiar with mobile technologies, a printed questionnaire was delivered by post and collected through a neighbourhood collection point designated by the researchers.

The survey was conducted in May and June 2023, approximately 5 months after the peak of COVID-19 infection in December 2022. Only participants aged 25-54 years were included to reduce potential age-related biases (18, 19). Participants who were not diagnosed as positive (hospital or self-tested antigen) in China after December 2022 were excluded. Participant’s participation in the survey implied their consent, which was stated in the promotional materials and on the survey’s front page. The study was approved by the Macau University of Science and Technology Medical Ethics Committee (MUST-HSS-20230505001).

### Sample size

Based on the literature, it was estimated that the prevalence of unresolved COVID-19-related symptoms at about 4 months among COVID-19 survivors was 45% (20). Using the prevalence-based calculation of the sample size with a precision of 5% and a confidence interval of 95% was estimated that a sample of at least 380 would be required to provide sufficient power for the study (21). Given an estimated 20% of invalidation rate, the final required sample size was estimated to be about 460.

### Measures

Mental Health status was assessed using the Depression, Anxiety, and Stress Scale (DASS) (22). The DASS designed to assess the severity of symptoms related to depression, anxiety, and stress. Participants are asked to rate the extent to which they have experienced each symptom over the past week using a 4-point Likert scale ranging from 0 (Did not apply to me at all) to 3 (Applied to me very much or most of the time). The final scores will be for depression (7 items, range 0-21), anxiety (7 items, range 0-21) and stress (7 items, range 0-21).

The LC symptomology was assessed by the Long-COVID Symptoms Checklist of 15 questions on symptoms commonly identified with patients with LC symptoms. Patients were asked if the symptoms persisted for at least two months after diagnosis of COVID-19, or if they had developed any new symptoms that had been present for a minimum of two months following their initial diagnosis. In this study, LC symptomatology was defined as having at least one of the symptoms reported on the Symptom Checklist. The validity of the Long-COVID Symptoms Checklist was tested by 10 global experts from cardiology, respiratory medicine, intensive care, and internal medicine. The content validity of the scale was calculated using the item-level content validity index (I-CVI) and the average scale-level content validity index (S-CVI/Ave). An I-CVI ≥ 0.78 and an S-CVI/Ave ≥ 0.90 were considered acceptable (23). Additionally, to evaluate the internal consistency of the items measuring Long-COVID with respect to the underlying construct, Cronbach’s α reliability coefficient was computed using SPSS software (SPSS 26.0: SPSS; Chicago, IL, USA). The I-CVI of each item of the Checklist ranged from 0.8 to 1.0, and the S-CVI/Ave was 0.87 with an I-CVI ≥ 0.78 and an S-CVI/Ave ≥ 0.90 considered acceptable (23). For internal consistency, Cronbach’s alpha coefficient was calculated as 0.80 with a value exceeding 0·7 regarded as acceptable (24).

To assess the burden of LC symptoms, patients were asked to rate each symptom on the Long-COVID Symptom Checklist the degree to which it interfered with their daily activities (including work and household duties) on a 5-point Likert scale ranging from 0 (Not at all) to 4 (Very much). The total perceived burden was assessed using the total score summing the responses on each item.

Resilience was examined by using the shortened version of the Connor-Davidson Resilience Scale (CD-RISC2) (25). The scale is based on two items that they believed captured the essence of resilience conceptually from the 25-item the Connor-Davidson Resilience Scale. It uses 5-point Likert-type response scale, ranging from “not at all” (0 points) to “almost completely” (4 points) (26). Participants rated each item based on their experiences during the past month. Higher scores indicate higher levels of resilience.

Social Support was assessed using The Duke-UNC Functional Social Support Questionnaire (DUFSS), which is a multidimensional social support scale consisting of 8 items used to measure an individual’s perceived social support (27). The DUFSS is a reliable and valid self-report tool that has been used in research with medical patients (28). Scores range from 8 to 40, with higher scores indicating greater perceived social support.

Other information collected included demographics, pre-COVID-19 health status, vaccination history, date of their first COVID-19 symptom onset, symptoms in the acute infection phase, hospitalisation during the acute symptomatic period, and how they confirmed their COVID-19 infection.

### Statistical analysis

The statistical analyses were performed in STATA 15.1 (StataCorp LLC, TX, USA). Descriptive analyses were performed to examine the participants’ demographic and health-related profiles, the prevalence of symptoms, and scores on psychological scales. Continuous variables are expressed as the mean (SD), while binary and categorical variables are presented as counts and percentages. Bivariate analyses were conducted between LC symptomology, demographic, health-related and other study variables, and the three mental health variables, namely depression, anxiety, and stress separately. For the selection of variables to be included in further multiple linear regression analyses, a criterion of p<0.1 was applied. For the association between LC symptomology and mental health, multiple linear regression analyses were employed since the raw scores of the DASS were used as the outcome measures. The possible moderating effect of resilience and social support between LC symptomology and poor mental health was checked by testing their corresponding interaction terms in the regression models. The analyses of the relationship between the perceived burden of LC and mental health were only conducted in the subgroup of patients with LC symptomology; namely, patients responded positively to the question “experienced lingering symptoms at least lasting for 2 months that cannot be explained except due to the infection.”. A similar approach to the aforementioned analyses for the association between LC symptomology and mental health was adopted. A type I error rate of 5% was used for all 2-tailed hypotheses testing for independent variables and 1% for the interaction terms.

## Results

In total, 781 patients tested positive with COVID-19 were recruited. Of these 482 respondents provided useful information for data analyses, after removing 299 questionnaires having inconsistent answers on geographical information or confirmation date of infection. A comparison between the respondents and non-respondents indicated no differences in demographic variables. **Table 1** presents patients’ demographics, basic health information, and scores on mental health measures. Of the total 482 respondents, there were 195 (40.5%) males, with 374 (77.6%) receiving three or more doses of vaccine, and the most commonly received vaccine was the domestically produced inactivated vaccine (66.8%), followed by the domestically produced recombinant protein vaccine (30.5%). The most prevalent symptom of acute-phase infection reported by participants was fever (87.1%), followed by muscle and joint pain (56.0%), cough (47.9%), fatigue (42.7%), headache (41.9%), ENT abnormalities (25.1%), and finally shortness of breath (12.4%). Seven (n=7, 1.5%) of the respondents reported pre-morbid mental health problems, and 6 (1.2%) were hospitalised during the acute phase of the infection, with half (50%) of these hospitalised patients receiving treatment in the ICU. In terms of the outcome measures of the study, namely the mental health status, 61 (12.7%) exhibited symptoms of severe to extremely severe depression symptoms (mean=15.9, s.d=4.7), 26.8% (n=129) anxiety (mean=12.2, s.d=4.8), and 25.1% (n=121) stress (mean=17.2, s.d=3.8).

**Table 1:** Descriptive information on the demographics, premorbid health, vaccination, hospitalisation, resilience, social support, and mental health symptoms (N=482).

	Overall
**N**	482
Age	
25-34 years	130 (26.97)
35-44 years	153 (31.74)
45-54 years	199 (41.29)
Sex	
Male	195 (40.46)
Education	
Below bachelor’s degree	179 (37.1)
Smoking Status	
Yes	69 (14.32)
Alcohol Drinking	
Yes	113 (23.44)
Comorbidity of Mental Disorders	
Yes	7 (1.45)
Number of Vaccination	
Less than 3	108 (22.41)
Vaccination Type	
Inactivated	322 (66.80)
Recombinant	147 (30.50)
Others	13 (2.70)
Symptoms in acute phase	
Exhaustion	
Yes	206 (42.74)
Headache	
Yes	202 (41.91)
Dyspnea	
Yes	60 (12.45)
Fever	
Yes	420 (87.14)
Coughing	
Yes	231 (47.93)
Ear, Nose and Throat	
Yes	121 (25.10)
Muscles and Joints Pain	
Yes	270 (56.02)
Hospitalised	
Yes	6 (1.24)
ICU	
Yes	3 (0.62)
**Resilience (mean (SD))**	4.90 (1.94)
**SocialSupport (mean (SD))**	27.37 (7.21)
Depression (mean (SD))	
None/mild/moderate	421 (87.34)
Serious/Extreme serious	61 (12.66) 15.90 (4.74)
Anxiety (mean (SD))	
None/mild/moderate	353 (73.24)
Serious/Extreme serious	129 (26.76) 12.17 (4.83)
Stress (mean (SD))	
None/mild/moderate	361 (74.90)
Serious/Extreme serious	121 (25.10) 17.22 (3.77)

The results of LC symptomology were summarised in **Table 2**. As shown, among all respondents, 25.5% had symptoms that persisted for more than two months and could not be explained by causes other than COVID-19 infection. In addition, the top five common symptoms were postexertional malaise (22.2%), fatigue (21.2%), brain fog (14.3%), sleep (11.6%), shortness of breath (11.4%), and palpitations (10.2%).

**Table 2:** The frequency (%) of Long-COVID symptoms in the sample.

Long-COVID Symptoms	Frequency (%)
Dyspnea	55 (11.41)
Fatigue	102 (21.16)
Postexertional Malaise	107 (22.20)
Brain Frog	69 (14.32)
Sleep problems	56 (11.62)
Cough	43 (8.92)
Chest Pain	19 (3.94)
Sore Throat	23 (4.77)
Anosmia	46 (9.54)
Headache	16 (3.32)
Palpitations	49 (10.17)
Muscles and Joints Pain	40 (8.30)
Digestive Abnormalities	30 (6.22)
Hair Loss	44 (9.13)
Allergies	22 (4.56)
Risk of Long-Covid	123 (25.52)

The unadjusted bivariate relationships between LC symptomology, other study variables, and depression, anxiety, and stress were summarised in **Table 3**. As shown, a few variables were related to the mental health variables. LC symptomology, resilience, social support, and premorbid mental health problems were significantly related to all three aspects of mental health of interest. In addition, education level was significantly associated with stress and marginally related to anxiety. All other variables were insignificantly related to the mental health variables with a p-value much greater than 0.1. Hence, they were not included in further analyses. The results obtained from the multiple linear regression analyses were presented in **Table 4**. After adjusting for other variables in the model, the LC symptomology was still positively and significantly associated with depression (t=2.09, p=0.037) and anxiety (t=4.51, p<0.001), but not stress. The regression coefficient of LC symptomology was 1.13 (s.e. = 0.54) for depression suggesting a difference in the depression score of more than 1 unit between patients with LC symptomology and those without. The regression coefficient of LC symptomology for anxiety was 2.35 (s.e. = 0.52) indicating a difference of more than 2.3 units in the anxiety score between groups. In terms of the interaction terms between LC symptomology and resilience, as well as social support, the results indicated that none were significant even at the 5% types I error rate.

**Table 3:** Unadjusted association between Long-Covid Symptomology, demographics, premorbid health, vaccination, hospitalisation, resilience, social support, and mental health symptoms.

Variables	Depression	Anxiety	Stress
Age	F_(2, 479)_ = 0.65, p=0.521	F_(2, 479)_ = 0.02, p=0.984	F_(2, 479)_ = 1.98, p=0.140
Sex	t_480 = _1.44, p=0.152	t_480 = _1.34, p=0.181	t_480_= -0.12, p=0.907
Education level	t_480_= -0.20, p=0.846	t_480_= -1.90, p=0.059	***t_480_= -2.12, p=0.035***
Premorbid mental health problem	t_480_***= -3.90, p<0.001***	t_480_***= -4.47, p<0.001***	***t_480_= -2.04, p=0.042***
Smoking	t_480 = _0.12, p=0.908	t_480 = _0.54, p=0.590	t_480 = _0.42, p=0.677
Drinking	t_480 = _0.07, p=0.946	t_480_= -0.57, p=0.569	t_480_= -0.11, p=0.915
Number of vaccination	t_480 = _1.419, p=0.234	t_480_= -0.43, p=0.670	t_480 = _0.01, p=0.996
Vaccine type	F_(2, 479)_ = 1.91, p=0.150	F_(2, 479)_ = 1.01, p=0.364	F_(2, 479)_ = 1.41, p=0.244
Hospitalisation	t_480_= -0.45, p=0.655	t_480_= -0.42, p=0.672	t_480 = _0.77, p=0.444
Resilience	***r= -0.32, p<0.001***	***r= -0.32, p<0.001***	***r= -0.27, p<0.001***
Social support	***r= -0.29, p<0.001***	***r= -0.26, p<0.001***	***r= -0.19, p<0.001***
Long-COVID Symptomology	t_480_***= -2.77, p<0.001***	t_480_***= -5.22, p<0.001***	t_480_***= -2.41, p=0.017***

The bold values indicated significance of p value (p<0.05).

**Table 4:** Result of the adjusted association between the Long-COVID Symptomology and mental health (N=482).

Variables retained in the model	Depression				Anxiety				Stress			
	β	s.e.	t	p-value	β	s.e.	t	p-value	β	s.e.	t	p-value
Risk of Long-COVID	1.13	0.54	2.09	0.037	2.35	0.52	4.51	<0.001	0.87	0.56	1.55	0.122
Premorbid mental health problem	6.26	1.96	3.20	0.001	7.25	1.89	3.83	<0.001	2.70	2.05	1.23	0.188
Education level	–	–	–	–	1.28	0.47	2.70	0.007	1.46	0.51	2.85	0.005
Resilience	-0.62	0.14	-4.33	<0.001	-0.63	0.14	-4.60	<0.001	-0.63	0.15	-4.25	<0.001
Social support	-0.12	0.04	-3.12	0.001	-0.10	0.04	-2.70	0.007	-0.06	0.04	-1.69	0.092
Model statistics	F_(4, 477)_ =21.06, p<0.001, R^2 = ^15.0%				F_(5, 476)_ =17.09, p<0.001, R^2 = ^15.2%				F_(5, 476)_ =10.62, p<0.001, R^2 = ^10.0%			

For the relationships between the perceived burden of LC symptoms and mental health, the results were presented in **Table 5**. As shown, after adjusting for variables including premorbid mental health problem, education level, resilience, and social support, positive and significant associations were found between the perceived burden of LC and depression, anxiety, and stress. The regression coefficients of the perceived burden of LC for depression and stress were 0.38 (s.e. = 0.07) and 0.35 (s.e. = 0.08) respectively, indicating, on average, for each increase in 1 unit of perceived burden measure there was a corresponding increase of 0.38 units in the depression and 0.35 in the stress scores. Among the three mental health variables, anxiety had the largest increase in the score with an increase of each unit of perceived burden of LC there was an increase of 0.54 units (s.e. = 0.07, t=8.24, p<0.001). These results suggested a positive dose-response relationship between the perceived burden of LC symptoms and mental health problems. As in the previous analyses, none of the interaction terms between perceived burden and resilience, as well as social support were significant.

**Table 5:** Result of the association between the perceived symptom burden of Long-COVID and depression, anxiety, and stress (N=123).

Variables retained in the model	Depression				Anxiety				Stress			
	β	s.e.	t	p-value	β	s.e.	t	p-value	β	s.e.	t	p-value
Burden of Long-COVID	0.38	0.07	5.45	<0.001	0.54	0.07	8.24	<0.001	0.35	0.08	4.37	<0.001
Premorbid mental health problem	2.59	3.20	0.81	0.420	3.95	3.01	1.31	0.192	0.82	3.60	0.23	0.821
Education level	–	–	–	–	0.73	0.47	0.89	0.373	1.79	0.98	1.82	0.071
Resilience	-0.51	0.25	-2.03	0.045	-0.72	0.24	-3.03	0.003	-0.64	0.28	-2.24	0.027
Social support	-0.15	0.07	-2.21	0.029	-0.11	0.06	-1.73	0.085	-0.06	0.08	-0.75	0.454
Model statistics	F_(4, 118)_ =13.55, p<0.001, R^2 = ^31.5%				F_(5, 117)_ =21.38, p<0.001, R^2 = ^47.7%				F_(5, 117)_ =7.46, p<0.001, R^2 = ^24.2%			

## Discussion

This study aimed to investigate the prevalence of common symptoms of LC, and the propensity of LC in a community sample of COVID-19 patients. It also aimed to examine the relationship between LC symptomology and the mental health status, as well as the relationship between the perceived symptom burden of LC and mental health problems. The results suggested that patients exhibited the overall LC symptomology was about 25% among patients who had tested positive for COVID-19 after December 2022. Postexertional malaise and fatigue were the most prevalent LC symptoms, accounting for 22.2% and 21.2% of all LC patients, followed by cognitive impairment including brain frog (14.3%), and sleep problems (11.6%). The results further suggest a positive and significant association between LC symptomatology and poor mental health, particularly depression and anxiety. In terms of the perceived burden of LC symptoms, it is related to the mental health of patients also demonstrating a positive dose-response relationship with depression, anxiety, and stress.

The results on symptomatology, in general, are consistent with those reported in the literature, though some minor discrepancies are found in other less commonly reported symptoms, such as headaches and muscle aches that have been ranked less prevalent in the current study (29, 30). Furthermore, some differences in the prevalence of symptoms have also been identified compared to previous studies on COVID-19 patients one-year post-infection in China (12, 31). In Zhang’s study, the prevalence of muscle and joint pain was 11.8%, whereas in the current study, it was 8.3% (12). While the prevalence of brain fog and palpitations were 14.3% and 10.2% in this study, they are higher than that reported in the literature of 2.2% and 5.8% (31) This study also found that patients with LC symptomology scored significantly higher in depression and anxiety, but not stress. In terms of the propensity of these symptoms, these patients reported postexertional malaise, fatigue, brain fog, shortness of breath, and insomnia, as the most common symptoms, indicating that these symptoms had a greater impact on people’s lives and work. In the present study, postexertional malaise (PEM), a distinguishing feature of chronic fatigue syndrome (CFS) (32), was most prevalent and reported to be the most burdensome among other symptoms identified as post-COVID-19 sequelae. There could be many explanations for this result. One possible reason may relate to the potential physiological responses for the infection in different body parts including the brain, such as neuroinflammation (33). This has highlighted how the chronic and debilitating fatigue associated with CFS can significantly impact patients’ work productivity and quality of life, posing psychological distress (34). Moreover, symptoms related to CFS are relatively difficult to diagnose in hospitals to receive appropriate rehabilitation recommendations (35).

For the relationship of LC and mental health, the changes to routine happened due to pandemic, such as financial instability, social distance and mask wearing, have been challenging people’s psychological needs that previously fulfilled for about 3 years (36–38). Moreover, as for patients developed LC symptoms, they might experience additional symptoms related to brain function leading to a worse mental health outcome compared to those who recovered well from COVID-19 infection (39). The perceived burden of LC symptoms further exhibits the impact of the disease on the mental health of patients. There could be many reasons attributing to the interplay between the actual severity of the physical illness, treatment demands and individuals’ perceptions of their ability to adapt (40). According to the Burden of Treatment Theory (14), the perceived disease burden in LC patients may be aggregated by the difficulties of managing the symptoms. Overall, medical expenses on the treatment, which was estimated at $9,000 per person annually, could be a stress for many people (41). Moreover, the increased stringency of containment measures during the pandemic resulted in unemployment also exerted additional pressure on patients (42). As a result, patients who would like to retain their jobs are more likely to keep working and not take any medical leaves, thus becoming an additional layer of burden.

The findings highlight the need to provide mental health support for those severely affected by COVID-19 infection, particularly those who developed LC symptoms. In 2020, the National Health Commission of China issued “Rehabilitation Program for Discharged COVID-19 Patients” (43) recommending respiratory training, physical exercises, psychological support, and activities to regain daily living abilities to address residual respiratory, physical, and psychological dysfunctions like cough, fatigue, and anxiety that may persist post-hospitalisation. To date, the treatment strategies for LC in designated clinics or outpatient services mainly focused on symptom relief (44), with both Western and Chinese medicine focusing on physical rehabilitation (45, 46). In terms of mental health services in general, and particularly for patients with LC symptoms, it is a lacking area. China has very few numbers of mental health professionals relative to its large population (47). There is also a lack of mental health rehabilitation services, and the utilisation of telemedicine is also low mainly due to the limited access to facilities (47). With such limited resources, well-designed and validated preventive programs using a mHealth approach for early detection, intervention, treatment and management are urgently needed for the patient population with LC symptoms (48, 49).

### Limitations

There are several limitations to our study that should be noted. First, our sample was limited to individuals who self-reported LC symptoms, which may not be representative of all patients with LC symptoms. Out of the total sample, only 7 patients reported pre-morbid mental problems, which could limit the generalisation of correlation found between pre-morbid mental problems and mental illness severity after COVID-19 infection. Second, due to the study design being a cross-sectional survey, it would be difficult to draw any conclusion on the causality of the results obtained. Longitudinal studies could undoubtedly provide more powerful data for interpreting causality. While many other countries have established app-based COVID-19 syndromic surveillance systems (50, 51), a similar tracking platform has not yet been deployed in China (52). In addition, our study was conducted in a single city in the northern part of China. This may not be representative of the whole country due to possible geographical differences across the land, thus making it difficult to generalise the findings. For better evidence of the effect of LC on the mental health of infected patients’ studies of a stronger design, such as a cohort study, should be conducted. Furthermore, the study should also be expanded to a wider geographical region with more diverse populations.

However, despite these limitations, our study also has important contributions. To our knowledge, this is one of the first studies to investigate the impacts of perceived LC burden on mental health in China through a standardised assessment procedure. While larger and more rigorous studies are still needed, our findings provide valuable preliminary insights into how LC may be associated with increased mental illness severity.

## Conclusion

The present study provides evidence that LC symptomology is associated with mental health problems of patients and that there is a dose-response relationship between the burden of LC symptoms and mental health problems. Long-term management and rehabilitation are essential in providing support to these patients. Continued investigation of how LC may influence mental well-being may also provide insights to guide targeted interventions and management strategies.

## Data availability statement

The raw data supporting the conclusions of this article will be made available by the authors, without undue reservation.

## Ethics statement

The informed consent and study protocol was confirmed by the Macau University of Science and Technology Medical Ethics Committee (MUST-HSS-20230505001).

## Author contributions

YL: Conceptualization, Formal analysis, Methodology, Visualization, Writing – original draft. LL: Formal analysis, Supervision, Writing – review & editing. YX: Conceptualization, Supervision, Writing – review & editing. ZQ: Investigation, Validation, Writing – original draft. YZ: Investigation, Validation, Writing – original draft.
